# Temperature Affects the Tripartite Interactions between Bacteriophage WO, *Wolbachia*, and Cytoplasmic Incompatibility

**DOI:** 10.1371/journal.pone.0029106

**Published:** 2011-12-15

**Authors:** Sarah R. Bordenstein, Seth R. Bordenstein

**Affiliations:** Department of Biological Sciences, Vanderbilt University, Nashville, Tennessee, United States of America; Centro de Pesquisas René Rachou, Brazil

## Abstract

*Wolbachia* infections are a model for understanding intracellular, bacterial symbioses. While the symbiosis is often studied from a binary perspective of host and bacteria, it is increasingly apparent that additional trophic levels can influence the symbiosis. For example, *Wolbachia* in arthropods harbor a widespread temperate bacteriophage, termed WO, that forms virions and rampantly transfers between coinfections. Here we test the hypothesis that temperatures at the extreme edges of an insect's habitable range alter bacteriophage WO inducibility and in turn, *Wolbachia* densities and the penetrance of cytoplasmic incompatibility. We report four key findings using the model wasp, *Nasonia vitripennis*: First, both cold treatment at 18 C and heat treatment at 30 C reduce *Wolbachia* densities by as much as 74% relative to wasps reared at 25 C. Second, in all cases where *Wolbachia* densities decline due to temperature changes, phage WO densities increase and inversely associate with *Wolbachia* densities. Heat has a marked effect on phage WO, yielding phage densities that are 552% higher than the room temperature control. Third, there is a significant affect of insect family on phage WO and endoysmbiont densities. Fourth, at extreme temperatures, there was a temperature-mediated adjustment to the density threshold at which *Wolbachia* cause complete cytoplasmic incompatibility. Taken together, these results demonstrate that temperature simultaneously affects phage WO densities, endosymbiont densities, and the penetrance of cytoplasmic incompatibility. While temperature shock enhances bacteriophage inducibility and the ensuing bacterial mortality in a wide range of medically and industrially-important bacteria, this is the first investigation of the associations in an obligate intracellular bacteria. Implications to a SOS global sensing feedback mechanism in *Wolbachia* are discussed.

## Introduction

The obligate intracellular bacteria, *Wolbachia*, live in a majority of arthropod species worldwide [1]. Like a matryoshka doll, *Wolbachia* themselves harbor an obligate intracellular, temperate bacteriophage [2], [3] that is present in up to 89% of sampled strains [4], [5]. The integrated prophage WO in the *Wolbachia* genome [6] can form free phage particles [7] in approximately 12% of *Wolbachia* cells reared in insects at room temperature [8]. While extensive work has been done to understand lysogeny and lytic induction in medically or industrially important bacteria, no studies have defined the parameters under which prophages are induced from obligate intracellular bacteria. This dichotomy stems from the observation that mobile genetic elements have only recently been subject to increasing study in obligate intracellular bacteria [3], [9]–[17].

A massive phage-mediated lysis of an intracellular symbiont could cause dramatic changes in densities and functions. Indeed, the penetrance of all of the major reproductive alterations caused by *Wolbachia*, including cytoplasmic incompatibility (CI), male-killing, parthenogenesis, and feminization, are correlated with bacterial densities, and such density effects are not confined to *Wolbachia* endosymbionts (reviewed in [18]). While host and bacterial factors regulate *Wolbachia* densities *in vivo*, there are well-known environmental variables (e.g., temperature, antibiotics, and host age) that can decrease *Wolbachia* densities and functions across many species [18]–[21]. Intriguingly, some of these same variables induce viron formation and the lytic lifecycle of temperate bacteriophage in free-living bacteria. We thus hypothesize that environmental stresses can cause the reduced *Wolbachia* densities by triggering the induction of WO in *Wolbachia*. The rationale is based on the tenet that lysogenic phage “sense” stressed bacteria, thereby inducing lytic particles and a reduction of bacterial densities. The induction of lytic development in *Wolbachia* may be akin to a lambdoid global sensing feedback mechanism such as the SOS response [22]. Key SOS genes are present in the *Wolbachia* genome, including *recA*, *uvrA*, *uvrB*, *uvrD*, and a cI-like repressor; the *lexA* repressor is missing. While we are not testing the SOS mechanism per se, we aim to test if temperature triggers changes in the phage's titer and whether this variation correlates with variation in the densities and penetrance of *Wolbachia* functions following environmental stress.

Temperate phage WO produces virion particles that lyse *Wolbachia* cells, presumably with the aid of its annotated endolysin and patatin [3]. In the infected testes of *Nasonia vitripennis* wasps, *Wolbachia* cells with endogenous phage particle production show severe cellular defects including densely-stained degraded DNA, a collapsed inner membrane typical of the activity of lysins, and lysed membranes associated with the exit of the phage particle [8]. Moreover, quantitative analyses illustrate an inverse, predator-prey like association between relative densities of the phage and bacteria [8], [23]. Thus, WO particle production is coupled with *Wolbachia* cell lysis.

The *Nasonia* genus is one of a few tractable systems for understanding phage-*Wolbachia*-animal interactions because it has many features suited for genetic and functional studies. They have a short generation time, simple husbandry, fertile species hybridizations, a wide array of molecular markers [24] and three fully sequenced genomes [25]. *N. vitripennis* is normally coinfected by each of the two major insect-*W. pipientis* subdivisions, A and B [26]. These infections have been segregated in the lab into strains with single A or B infections [27]. Here, we describe a comprehensive set of experiments to test for the first time whether a common, environmental stress known to reduce *Wolbachia* densities (temperature) also affects the densities of bacteriophage WO in the A *Wolbachia*, termed WOVitA1 [16], and the ensuing reductions in *Wolbachia* densities and CI penetrance.

## Materials and Methods

### Insect Strains

Two *N. vitripennis* strains were used to test the effects of temperature on *Wolbachia* and bacteriophage WO densities. The experimental strain (12.1) is single A-infected and established in 1996 following the recovery of a double AB-infected isofemale strain (R511) from prolonged diapause [27]. The *Wolbachia*-uninfected control strain (12.1T) was derived from 12.1 by tetracycline treatment in 2008. Each strain was maintained under constant light at 25 C and raised on flesh fly pupae (*Sarcophaga bullata*).

### Temperature Treatment

Treatments of infected (12.1) and uninfected (12.1T) wasps were performed in 18 C, 25 C and 32 C incubators with constant light. Treatments were initiated during four developmental stages and continued through adulthood from: (i) two-hour old eggs, (ii) second-instar larvae, (iii) yellow-yellow pupae and (iv) adults. To collect each stage, virgin females were presented with four *S. bullata* host pupae for feeding and egg laying. After 48 hours, they were transferred to a new vial with one host for two hours. Parasitized hosts were incubated at 25 C and placed in treatment chambers when reaching designated development stages. Two-day old virgin males from each treatment were individually mated with uninfected virgin females at room temperature. Only those pairings where copulation was observed within 10 minutes were used. Males were immediately frozen at −80 C, and each female was provided two hosts and left undisturbed at 25 C. Adults were scored for sex ratios at death. Offspring numbers and sex ratios indicated that males reared at 32 C were affected by a reduction in fertility unrelated to *Wolbachia* infection status. Wasps were therefore tested at 30 C in a second experiment, with a separate 25 C control, because this temperature is within the development threshold of *N. vitripennis* and does not contribute to heat-related stress on immature development and fertility [28].

### Quantitative Analysis of *Wolbachia* and Bacteriophage WO Densities

Genomic DNA was extracted using the Gentra Puregene Tissue Kit (Qiagen, Valencia, CA) from single adults. Real-time quantitative polymerase chain reaction (RT-qPCR) was performed in a CFX96 system (Bio-Rad, Hercules, CA). Reaction volumes of 25 ul contained 12.5 ul of Bio-Rad SYBR Green Supermix, 8.5 ul sterile water, 1.0 ul of each 5 uM forward and reverse primer, and 2 ul target DNA in single wells of a 96-well plate (Bio-Rad). Selective amplification was performed on a small portion of the *Nasonia S6 Kinase* (*S6K*, 133 bp), *Wolbachia groEL* (*groEL*, 97 bp), and bacteriophage WO-B (*ORF7*, 125 bp) genes. Primers were designed for *NvS6K*: NvS6KQTF4 (5′-GGCATTATCTACAGAGATTTGAAACCAG-3′), NvS6KQTR4 (5′- CAAAGCTATATGACCTTCTGTATCAAG-3′). Primers for *groEL* and *ORF7* have been previously described along with PCR conditions [8]. Standard curves for each gene were constructed using a log10 dilution series of known amounts of PCR products from larger regions of the same gene: NvS6KQTF1 (5′-GGGAAAGCTTTATTTGATTCTTG-3′), NvS6KQTR3 (5′-GAGTAAGTCTGTGTATCATC-3), WGroF1 (5′-GGTGAGCAGTTGCAAGAAGC-3′), WGroR1 (5′-AGATCTTCCATCTTGATTCC-3′), PhgWOF (5′-CCCACATGAGCCAATGACGTCTG-3′), and PhgWOR (5′-CGTTCGCTCTGCAAGTAACTCCATTAAA-3′). All PCR reactions were performed in duplicate and included a melting curve analysis to check for primer-dimers and nonspecific amplification.

Both temperature experiments were analyzed using the BioRad CFX96 linear regression software. Small plate effects were observed by a trend of slightly elevated or reduced threshold cycle (Ct) levels for the same template standards used across all plates. Normalization was performed by applying correlation coefficient equations for each set of standards using GraphPad Prism Software (La Jolla, CA): (*S6K*: y = −3.41965x+36.1073), (*groEL*: y = −3.38355x+35.9005), and (*ORF7*: y = −3.36463x+35.1809) where x = log starting quantity of template DNA and y = Ct value.

### Statistics

Pairwise population comparisons of *Wolbachia* densities, phage WO densities in the cold-treatment experiment, and CI penetrance were conducted with the nonparametric Mann-Whitney U Test (MWU) at α = 0.05 (MiniTab v.12.23, State College, PA). Pairwise population comparisons of phage WO densities in the heat treatment experiment were conducted with the nonparametric Kolmogorov-Smirnov Test (KS) at α = 0.05 (MiniTab). The two-sample Kolmogorov-Smirnov test uses the maximal distance between cumulative frequency distributions of two samples as the statistic and is appropriate when there are ties in the data. However, the Mann-Whitney test takes the difference between mean ranks of the two samples as the statistic. Because the heat-treatment experiment elicited extreme variation in phage WO densities and ties in the analysis, we opted to use the more appropiate Kolmogorov-Smirnov test. Correlation coefficients were calculated using the nonparametric Spearman's rho (JMP v.5.0, SAS Institute Inc). Fisher's Exact Test at α = 0.05 (JMP) was calculated to determine the significance of association between categories of CI expression in cold and heat-treated *Nasonia*. Alternatively, the chi-square test at α = 0.05 (JMP) was calculated to determine the significance of association between categories of CI expression in 25 C control wasps due to the larger sample size. The nonparametric Kruskal-Wallis one-way analysis of variance at α = 0.05 (JMP) was conducted to determine the significance of family effects on CI penetrance.

## Results

### Effects of Cold and Hot Temperatures on *Wolbachia* Densities

**Figure 1:**
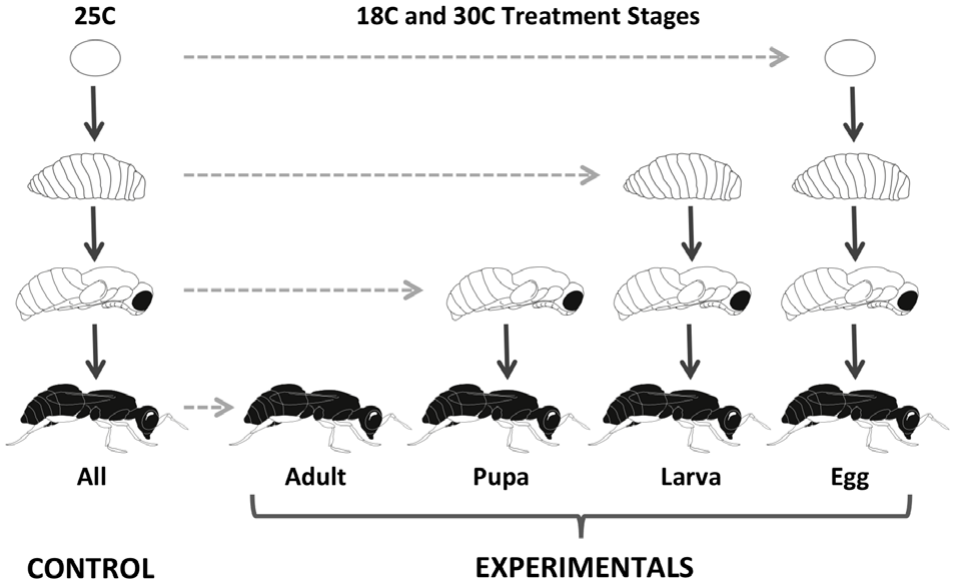
Schema of Experimental Design. *N. vitripennis* treatments were initiated during four developmental stages: (i) two-hour old eggs, (ii) second-instar larvae, (iii) yellow-yellow pupae and (iv) adults. Individual wasps were left undisturbed within *Sarcophaga* hosts for the remainder of development and experimental data was collected two days post-emergence from host.

To first characterize the effects of hot and cold temperatures on *Wolbachia* densities in *N. vitripennis*, we normalized absolute single gene copy counts from RT-qPCR of the *W. pipientis groEL* gene to that of the *N. vitripennis S6 kinase* (*S6K*) gene. Relative *Wolbachia* densities of two-day old adults were compared in two experiments: a cold temperature (18 C) vs. a control room temperature (25 C); and a hot temperature (30 C) vs. a control room temperature (25 C). Within each experiment, wasps were exposed to cold or hot temperatures for different development durations (e.g., egg to adult, larva to adult, pupa to adult, newly emerged adult) in order to evaluate which developmental durations are the most susceptible to temperature effects on microbial densities. Shorter durations of temperature shock were not found to affect microbial densities in preliminary experiments. Exposure began in five stages: two-hour old eggs, second instar larvae, pupae with yellow bodies and yellow eyes, or newly emerged adults and continued in all stages to two-day old adults (Figure 1).

**Figure 2:**
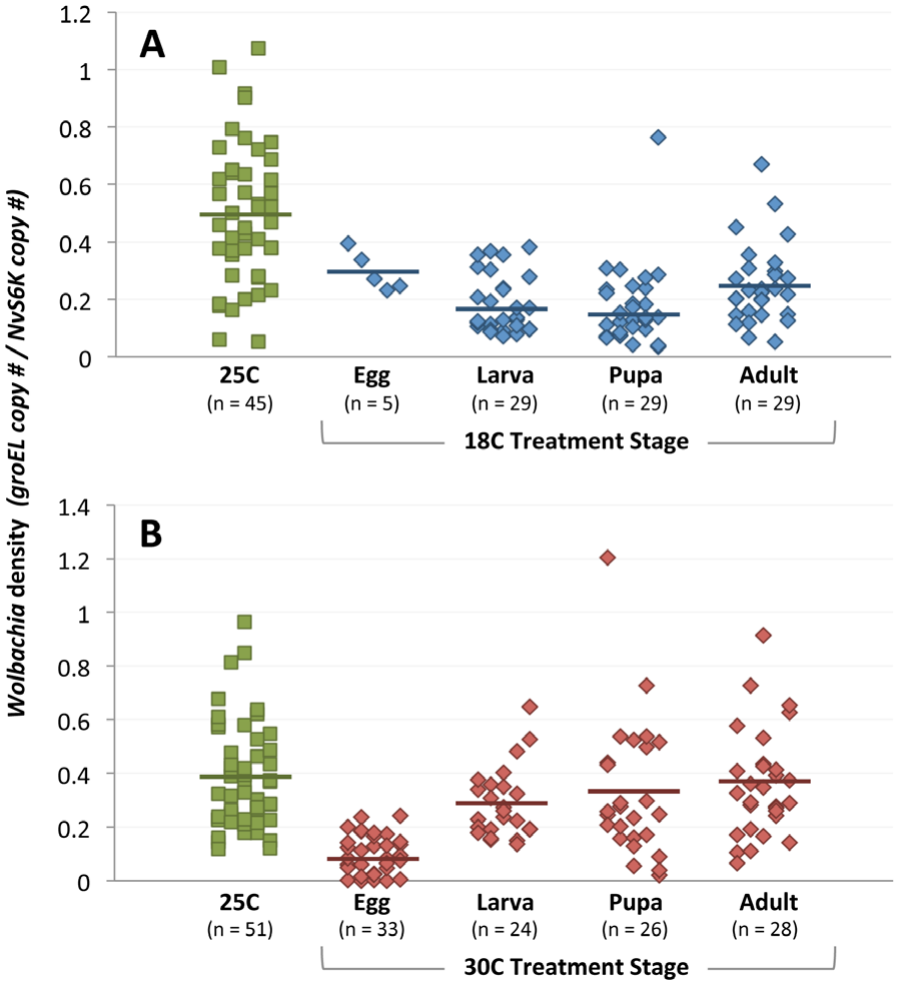
Effect of Temperature on *Wolbachia* Densities. *Wolbachia* densities are significantly lower in all stages of 18 C-treated wasps and in those that are exposed to 30 C from the egg stage of development. Points on the chart denote the number of *Wolbachia* genomes per *N. vitripennis* genome of an individual two-day old male after exposure to cold (A) or heat (B) treatment. Bars represent means.

In the cold treatment experiment, *Wolbachia* densities were significantly lower in 18 C-reared wasps than 25 C-reared wasps in all developmental stages (egg MWU, *P* = 0.0485; larval, pupal and adult MWU, *P*<0.0001) (Figure 2A). Comparing the relative effect of the different durations of cold treatment on densities also revealed that samples reared at 18 C from the larval (0.19±0.02; MWU, *P* = 0.0372) or pupal (0.18±0.03; MWU, *P* = 0.0094) stages had significantly lower mean densities ± standard error than wasps that were just exposed to 18 C during their first two days of adulthood (0.25±0.03). Development-specific differences to the the wasps reared at 18 C from the the egg stage could not be definitively determined owing to the small sample size of five individuals in this treatment.

In the heat experiment, *Wolbachia* densities were also significantly lower when wasps were reared at 30 C versus 25 C, but only in samples reared at 30 C from the egg stage. These wasps had densities (0.10±0.01) that were 3.0-fold lower than that of wasps reared at 25 C (0.38±0.03), as well as wasps reared under heat from the larval (0.29±0.03), pupal (0.33±0.05) and adult (0.36±0.04) stages (Figure 2B) (MWU, *P*<0.0001 for all comparisons). Thus, while cold treatment slightly reduces *Wolbachia* densities for any developmental duration of 18 C rearing, heat treatment markedly reduces densities in wasps reared just from the egg stage.

### Decreases in *Wolbachia* Densities Correlate with Increases in Phage Densities

To infer if *Wolbachia*'s bacteriophage was positively or inversely affected by the temperature stresses, we enumerated the number of phage gene copies of the *ORF7* gene by RT-qPCR and normalized this number to *Wolbachia* gene copies, forming a phage-to-bacteria ratio. We previously identified three different phage WO paralogs in the genome sequence of *N. vitripennis* A *Wolbachia* [16]. Thus, we expect a minimum ratio of three phages per bacterium during lysogeny.

**Figure 3:**
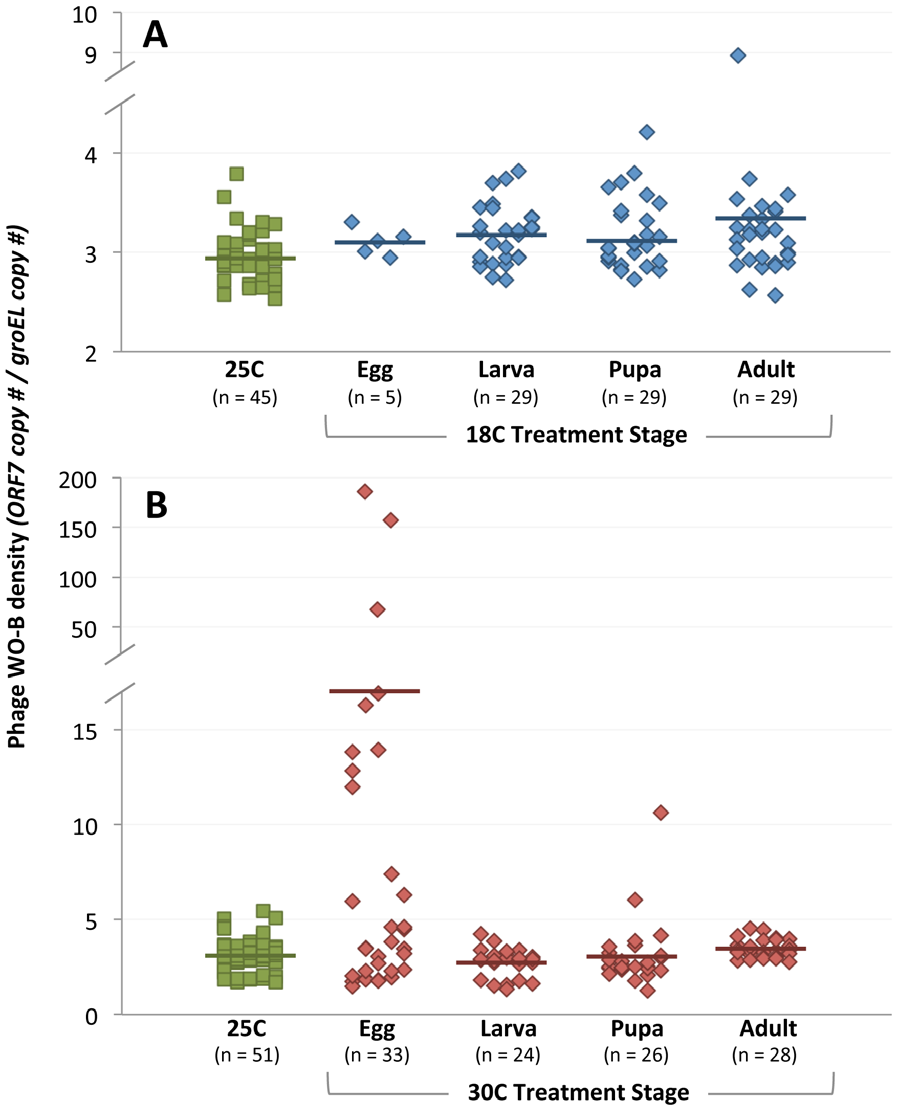
Effect of Temperature on Phage WO Densities. Phage WO densities are significantly higher in 18 C-treated wasps and in those that are exposed to 30 C from the egg stage of development. Points on the chart denote the number of phage WO *ORF7* genes per *Wolbachia* genome of an individual two-day old male after exposure to cold (A) or heat (B) treatment. Bars represent means.

Phage WO densities were moderately, but significantly higher in 18 C cold-reared samples from the larval (3.18±0.05; MWU, *P* = 0.0011), pupal (3.12±0.09; MWU, *P* = 0.0268), and adult (3.34±0.21; MWU, *P* = 0.0047) stages relative to samples reared at 25 C (2.96±0.04) (Figure 3A). There was a marginally insignificant difference between the cold-treated samples from the egg stage (3.11±0.06; MWU, *P* = 0.08) and the corresponding room temperature control, which we attribute to the small sample size of cold-treated individuals from this developmental stage (N = 5).

As expected for a lambda-like virus exposed to heat stress, the 30 C treatment of wasps reared from the egg stage had a drastic effect on phage densities. Mean phage WO densities (17.41±7.25) were over 500% higher than all the 25 C controls and the other developmental durations that were reared at 30 C (Figure 3B) (25 C control 3.15±0.12, KS, *P* = 0.007; larval 2.72±0.15, KS, *P* = 0.001; pupal 3.13±0.35, KS, *P* = 0.023; adult 3.43±0.09, KS, *P* = 0.006). This result is expected given that these samples were the only heat-treated individuals to also show a significant decline in *Wolbachia* densities. The exceptional increase in phage WO densities at 30 C is in part associated with significant variation between families in this treatment. We found that brothers within this treatment (and others) tend to harbor similar densities and express similar CI penetrance that can be significcantly different from sets of brothers derived from other families in the same treatment (see section below on Effects of Family). But when taken together, phage WO densities on average were always significantly higher when the *Wolbachia* densities were significantly lower in response to either cold or heat treatments. Furthermore, the lack of an effect on phage WO densities correlated with the lack of a significant effect on *Wolbachia* densities at both temperature treatments.

**Figure 4:**
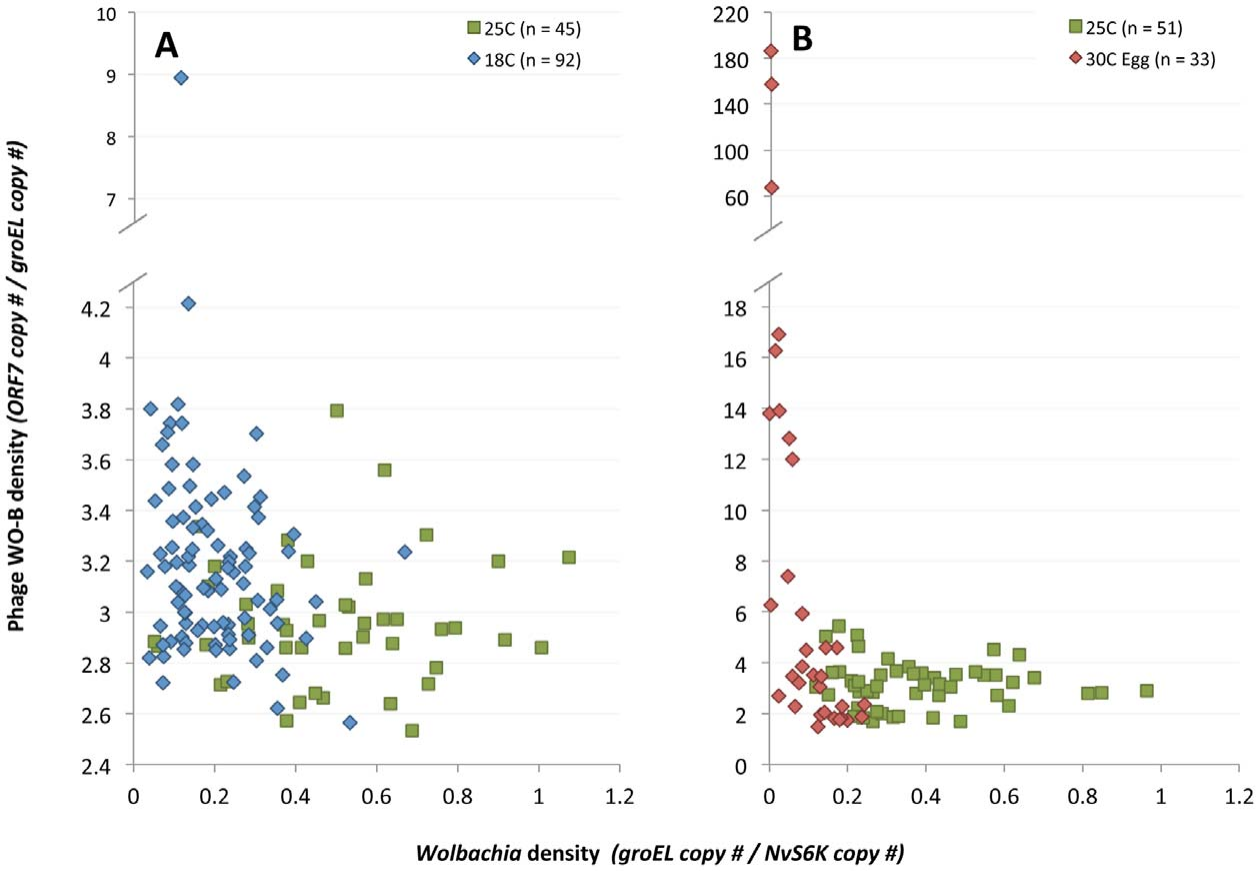
Inverse Association of Phage WO and *Wolbachia* Densities. Increase in phage WO density correlates with a decline in *Wolbachia* density. Points on the charts denote bacterial and bacteriophage densities in (A) individual cold-treated (blue) and (B) heat-treated (red) wasps compared to 25 C (green) controls.

To follow up on these observations, we tested the prediction from the ‘phage density model’ that phage densities inversely associate with *Wolbachia* densities based on the tenets that (i) bacteriophages are principally predators of bacteria and (ii) phage lytic development will inhibit bacterial replication or induce cell lysis [8]. Figure 4 confirms that relative phage densities are inversely correlated with *Wolbachia* densities for samples that were heat-treated since the egg stage (Spearman's rho = −0.7985, *P*<0.0001) and for all developmental stages that were reared in the cold (rho = −0.226, *P* = 0.0303). For instance, the highest phage densities (up to 186.2 WO genomes per *Wolbachia* genome) have among the lowest *Wolbachia* densities (0.0003 *Wolbachia* genomes per host wasp genome) and lowest absolute abundance of *Wolbachia* (less than 89.5 *groEL* copies) (see Table S1 for absolute copy numbers). Individuals reared at room temperature did not show significant associations between phage and *Wolbachia* densities as expected (Figure 4, cold experiment, rho = 0.0208, *P* = 0.892; heat experiment, rho = −0.0788, *P* = 0.5824). These findings specify that samples with significantly higher densities of phage WO have among the lowest *Wolbachia* densities.

### Effects of Temperature on CI and the Bacterial Dosage Model

In *N. vitripennis*, compatibility is measured according to percent females among progeny since only diploid females result from fertilized eggs in this haplodiploid species. Because normal sex ratios in *N. vitripennis* are female-biased, *Wolbachia*-induced incompatibility is expressed as the production of all-male (complete CI) or male-dominated (partial CI) families. In this experiment, complete CI refers to 0% female, partial CI refers to 1–49% females, and female-bias refers to any cross resulting in ≥50% females. The level of CI was determined by scoring percent females from single-pair matings between infected, temperature-treated males with uninfected females conventionally reared at 25 C.

**Figure 5:**
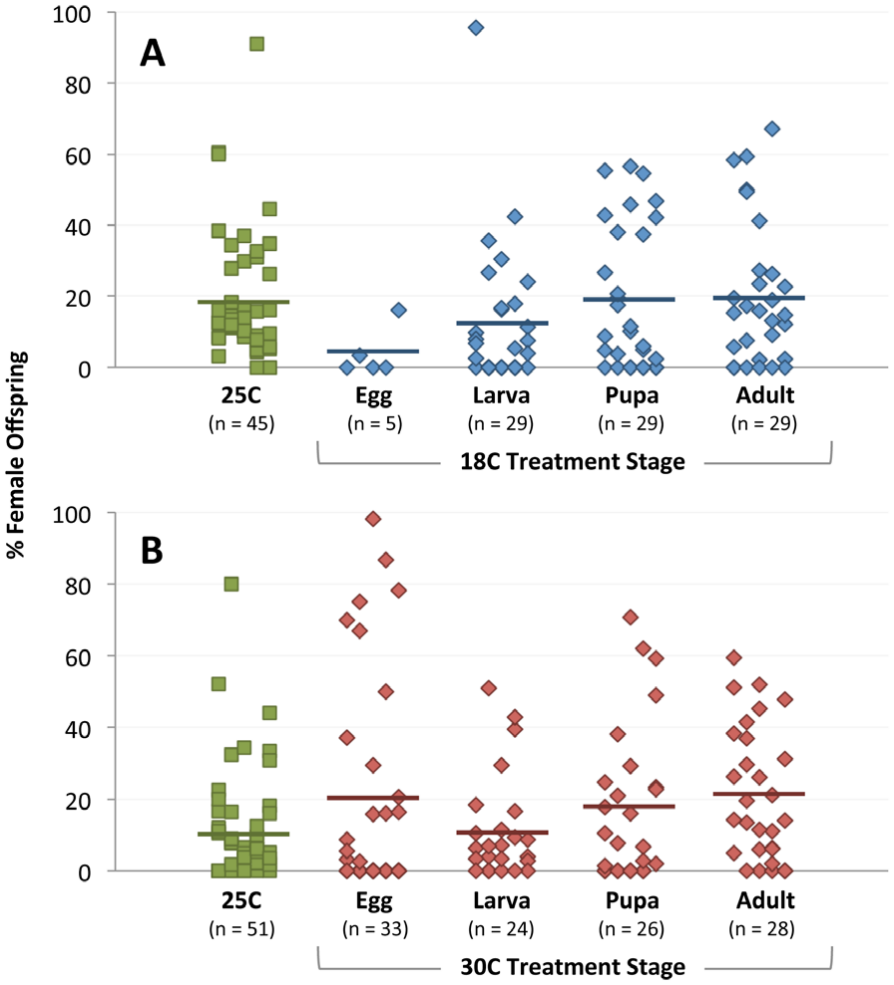
Effect of Temperature on CI Penetrance. CI penetrance is significantly greater when males are treated at 18 C (A) from the egg and larval stages and lower when males are treated at 30 C (B) from the egg stage. Points on the chart denote % female offspring in single-pair matings between uninfected (12.1T) females and infected (12.1) temperature-treated males. Bars represent means.

Both the cold and hot temperature experiments resulted in significant changes in CI penetrance, though surprisingly not in the expected directions based on the observed changes in *Wolbachia* densities. For example, CI penetrance (measured as a reduction in % females) was significantly greater in crosses with males that were reared at 18 C from the egg (MWU, *P* = 0.0139) and larval (MWU, *P* = 0.0058) stages relative to the 25 C control group (Figure 5A). The higher CI levels in these cold-reared wasps were unexpected as *Wolbachia* densities were shown to be significantly lower in the 18 C-reared wasps than 25 C-reared wasps in these stages (Figure 2). No effects were observed in the cold-treated pupal (MWU, *P* = 0.431) or adult (MWU, *P* = 0.8898) stages, which also had lower *Wolbachia* densities than the control group. Similarly unexpected, the heat treatment yielded significantly lower CI penetrance in 30 C-reared adults (MWU, *P* = 0.0139), but they did not yield different *Wolbachia* densities than the 25 C control group. Furthermore, the heat-reared samples from egg stage that did yield significantly lower *Wolbachia* densities did not yield different CI penetrance (egg, *P* = 0.6345), and no significant effects were observed in the remaining stages (larval, *P* = 0.8620; pupal, *P* = 0.4753) (Figure 5B).

According to the widely accepted “bacterial dosage” model of *Wolbachia*-induced CI [29], we expected to observe a correlation between bacterial density and the percentage of female progeny. Unidirectional incompatibility in *N. vitripennis* is strongly influenced by the density of *Wolbachia* in the male relative to that of the female and has been confirmed by multiple reports involving the same strains utilized in these experiments [8], [23]. Since temperature exposure affects *Wolbachia* densities in all cold treatments and in the egg heat treatment, we further explored this relationship by comparing the density of *Wolbachia* required to induce CI at different treatment stages relative to the control 25 C temperature.

By organizing families into either CI (<50% female, includes partial and complete CI) or female-biased (≥50% female) groupings, it was observed that the threshold density for *Wolbachia* to induce CI was significantly lower in 18 C cold-treated egg (0.30±0.03, MWU *P* = 0.0251), larval (0.18±0.02, MWU *P*<0.0001), pupal (0.18±0.03, MWU *P*<0.0001) and adult (0.27±0.03, MWU *P*<0.0001) stages than in the 25 C control (0.51±0.04). Similar to the trends above, the bacterial threshold was also significantly lower in the 30 C heat-treated egg stage (0.11±0.01) compared to the larval (0.29±0.03), pupal (0.36±0.05), adult (0.38±0.04) and 25 C (0.38±0.03) treatments (MWU, *P*<0.0001 for all comparisons).

**Figure 6:**
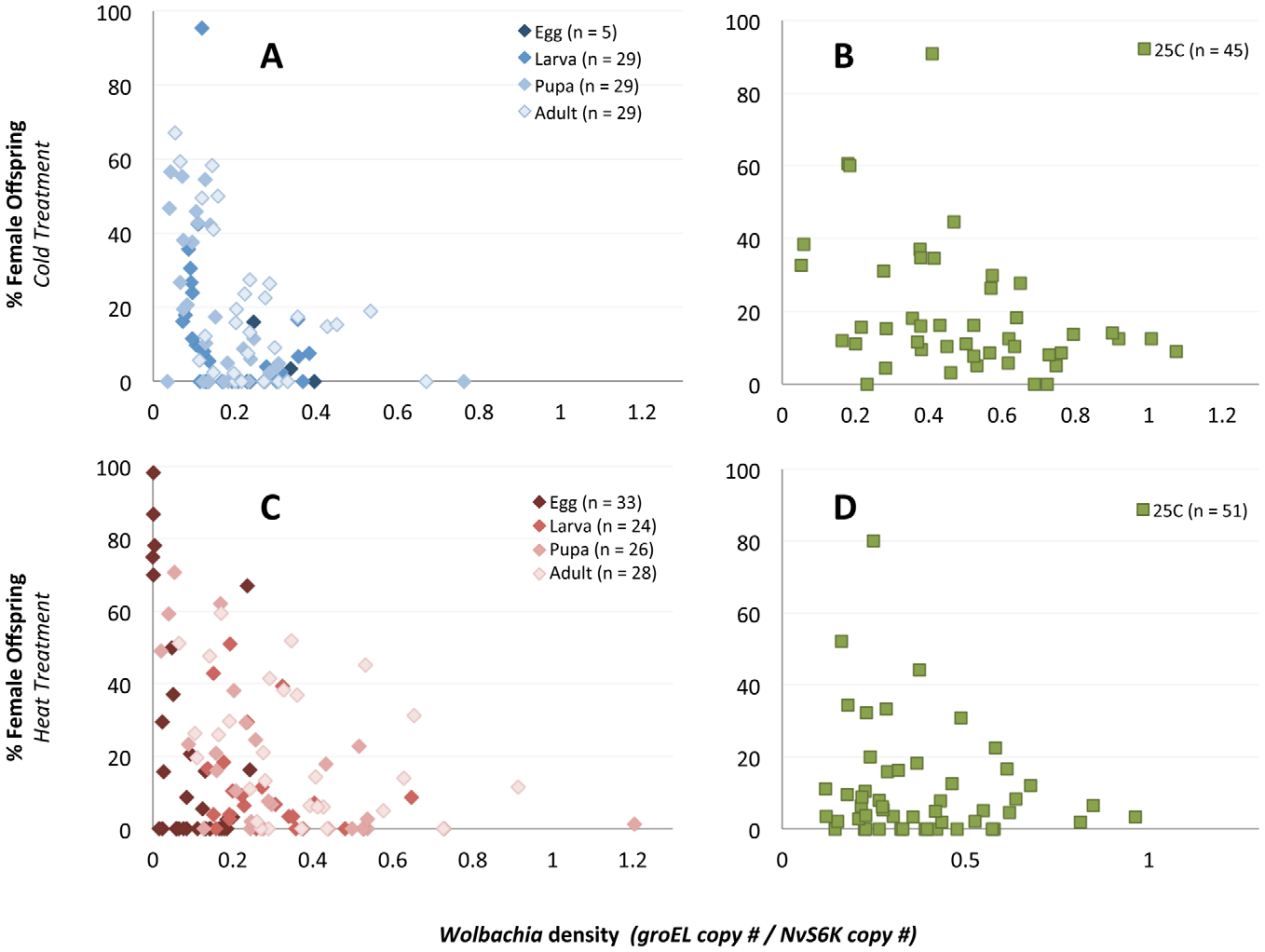
Correlation of *Wolbachia* Densities and CI Penetrance. There is a positive correlation between *Wolbachia* density and CI level for all temperature treatments. Points on the chart denote the *Wolbachia* density and % female offspring from single-pair matings between infected (12.1) *N. vitripennis* males exposed to 18 C (A), 25 C (B and D) or 30 C (C) temperature treatments and uninfected (12.1T) females.

*Wolbachia* densities are strongly correlated with the level of CI within each temperature treatment (Figure 6). The combined developmental stages of the cold treatment experiment revealed a stronger correlation between *Woblachia* density and percent females (rho = −0.4637, *P*<0.0001) compared to the 25 C control (rho = −0.3793, *P* = 0.0102). The egg stage of the heat treatment experiment also showed a slightly stronger correlation (rho = −0.4217, P = 0.0145) compared to the combined 25 C control group and all other heat treatment stages where *Wolbachia* densities were not affected by heat exposure (rho = −0.3372, P<0.0001).

**Figure 7:**
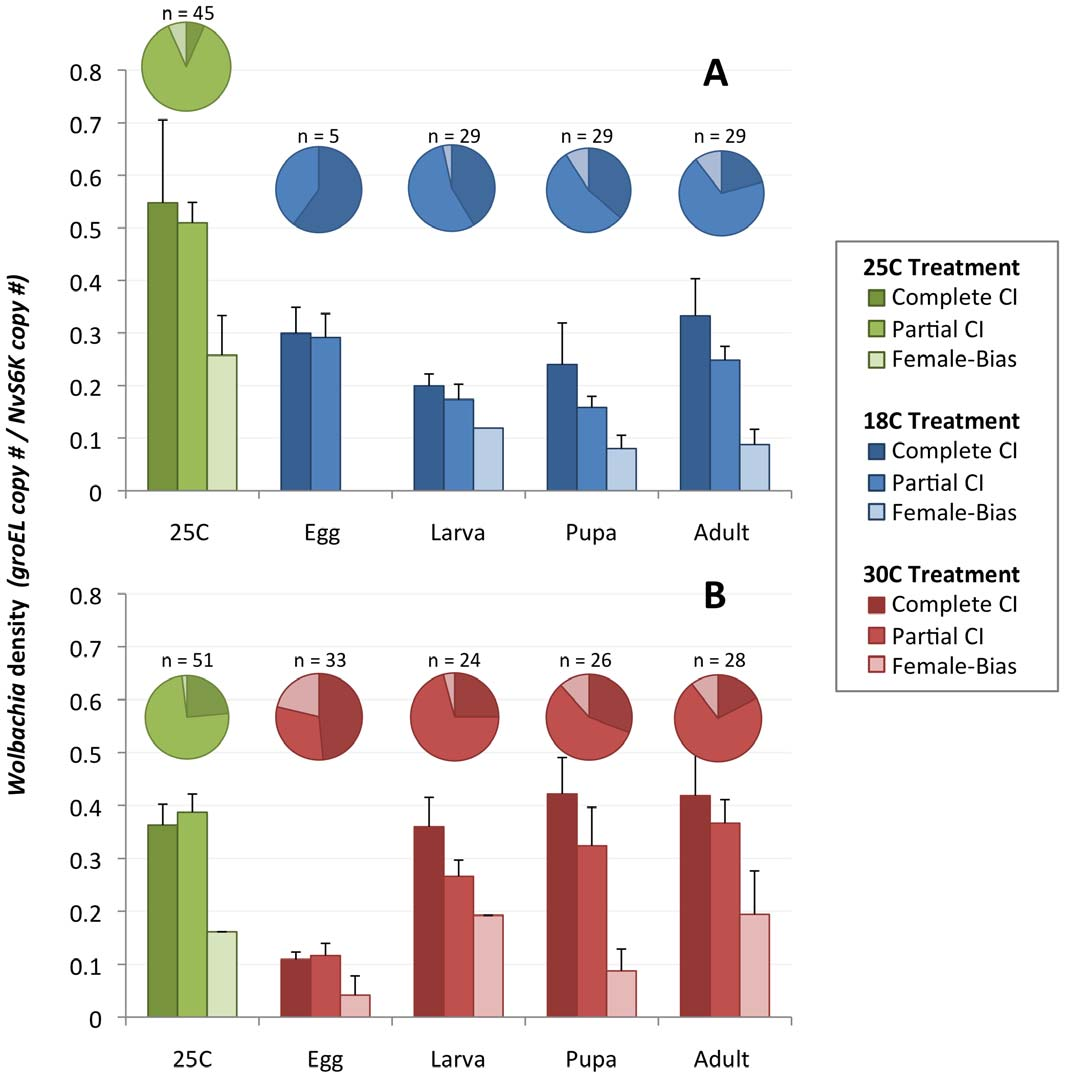
Relationships Between Temperature, *Wolbachia* Density and CI Level. Bars represent mean *Wolbachia* density ± standard error associated with complete CI, partial CI and female-bias for cold-treated (A) and heat-treated (B) *N. vitripennis* males. Pie charts represent the percentage of families expressing each CI level within the corresponding treatment stage and numbers above the charts indicate sample sizes. Room temperature controls are shown in green.

Figure 7 shows that the proportion of crosses expressing complete CI (0% female sex ratio) was significantly greater in wasps that were 18 C cold-reared from the egg (60.0%, Fisher's Exact Test, *P* = 0.0092), larval (41.0%, Fisher's Exact Test, *P* = 0.0006) and pupal (27.6%, Fisher's Exact Test, *P* = 0.0196) stages compared to the crosses from the 25 C control (6.67%). The proportion of crosses expressing complete CI was also greater in the case of males reared at 30 C from the egg stage (48.5%) compared to the 25 C control (23.5%, Chi-square, *P* = 0.0178). It is notable that the proportion of crosses expressing complete CI decreases correspondingly with the duration of the developmental period of temperature exposure. For example, a higher fraction of males reared at hot or cold temperatures from the egg stage express complete CI in comparisons to males reared at hot or cold temperatures from the larval stage, and so on.

### Effects of Family on CI, *Wolbachia* Densities and Phage WO Densities

During the course of these analyses, we noticed that brothers derived from the same mother tended to show similar levels of incompatibility. We therefore tested if there was a significant effect of family on incompatibility levels in the heat treatment. Family data was not recorded for the initial cold treatment and therefore not included in this analysis. We found that among *Wolbachia*-infected males, there was a significant effect of family in the following heat treatment stages: 25 C control (Kruskal-Wallis Test, *P* = 0.0256), 30 C egg (Kruskal-Wallis Test, *P* = 0.0007) and 30 C pupa (Kruskal-Wallis, *P* = 0.0242). These observations contrast with the same analyses in uninfected males in which there was only a significant effect of family in the 30 C egg treatment (Kruskal-Wallis Test, *P* = 0.0006). Because the effect of family was more pronounced in infected males, we then compared the *Wolbachia* densities of these families in pairwise tests. We found that brothers of the same family that had extreme low and high incompatibility levels (MWU, *P* = 0.0159) also showed inversely high and low *Wolbachia* densities (MWU, *P* = 0.0159), respectively. These effects, as expected, were inversely correlated with phage densities (MWU, *P* = 0.0317).

## Discussion

The majority of arthropod species harbor maternally-transmitted bacteria that are transmitted through the germ line. For these bacterial symbionts, within-host densities critically influence transmission efficiency to the next generation, the penetrance of beneficial or parasitic traits induced by the symbiont, and overall infection prevalence (reviewed in [18]). Among the most widespread germ line symbionts in animals, *Wolbachia* bacteria and their densities in hosts are primarily studied from three interrelated perspectives: (i) host, (ii) symbiont, and (iii) environment.

*Wolbachia* are, however, not immune to the lytic and lysogenic effects of their temperate phage WO [3], [8], [23]. In this study, we evaluated the interconnections between variation in temperature, *Wolbachia* and phage WO densities, and penetrance of cytoplasmic incompatibility. Stresses such as temperature are well-known environmental variables that decrease *Wolbachia* densities and expression of reproductive parasitism across host species [19]–[21], [30]. Intriguingly, heat shock is also one of the most common stimuli of inducing the lytic lifecycle of temperate bacteriophages. We therefore hypothesized that the underlying processes affecting *Wolbachia* depletion in response to temperature stress could be the inducibility of lytic phage and ensuing bacterial mortality. The temperature conditions examined in this study, 18 C, 25 C, and 30 C, are within the range of natural conditions for *Nasonia* parasitoid wasps.

We found that infected wasps reared at cold temperatures (18 C) for different developmental periods had lower *Wolbachia* densities than infected wasps reared at room temperature (25 C). The most marked difference, on average, was a 65% depletion in bacterial densities in the samples that were cold-reared from pupae to adults. The inhibitory effect of extreme cold temperatures (4 C) on *Wolbachia* densities in *Nasonia* diapause has been strategically utilized to generate cured strains or single-infected strains from double-infected strains [27], [31]. *Leptopilina heterotoma* wasps exposed to 18 C exhibit half as much *Wolbachia* as those reared at 26 C [32]. 18 C also mediates a reduction of endosymbiont densities for male-killing *Spiroplasma* bacteria in *Drosophila* [33]. Finally, increases and decreases of *Wolbachia* densities in eggs have been reported in cold treatments of *Drosophila bifasciata* [34] and *Drosophila simulans* [35], respectively.

Similarly we found that infected wasps reared at high temperatures (30 C) had lower *Wolbachia* densities than infected wasps reared at room temperature (25 C), but only for the samples heat-reared from the egg to adult stage. This significant depletion, on average, led to a 74% decline in bacterial densities. Effects of heat treatment on *Wolbachia* densities have been widely reported, and this research is the first time it has been studied in *Nasonia* wasps.

The variation in densities in response to cold and hot temperatures indicates that there can be considerable spatial, temporal, and seasonal differences in *Wolbachia* densities in nature. What causes the depletion of *Wolbachia* in temperature-treated samples? If the temperate phage WO does not play a role in the observed *Wolbachia* depletions, because the reduction is due to the extreme temperatures deterring bacterial replication, then the titers of the integrated prophage will be reduced in parallel with the *Wolbachia*. Statistically, we then expect a positive relationship between phage and *Wolbachia* densities. However, if the lytic form of phage WO underlies in part the bacterial decline during environmental stress, then the relative phage densities will increase in an inverse relationship to the *Wolbachia* densities [8]. At the lower temperature, we found that phage densities are significantly elevated across all four developmental periods in which *Wolbachia* densities were depleted. However, the highest increase in phage WO, on average, accounts for only a 12% expansion in phage densities. Such a small, but significant, increase probably does not explain the vast majority of the variation in bacterial densities in the cold treatment. Consistent with the basic observations of phage induction, cold temperatures do not appear to elicit a mass induction of prophage WO and most likely delay bacterial replication to cause reduced densities.

In contrast, heat treatment of wasps from the egg to adult stage caused, on average, a 552% increase in phage densities relative to the room temperature control. Thermoinducibility of temperate phages is a well-established rule. Curiously, high temperature treatment of the other developmental stages of the wasps did not induce a significant increase in phage densities. Why then is the treatment of eggs the most susceptible to causing major declines in *Wolbachia* and increases in phage WO? There are at least three interconnected explanations. First, *Wolbachia* cells could be more exposed to the effects of heat in eggs because they lack the protection afforded by being embedded in tissues and protected by the insect exoskeleton in the later developmental stages. Second, an immediate and strong depletion of *Wolbachia* in the egg stage may have a disproportionate effect on adult densities in comparison to larval, pupal, or adult stages since bacterial depletions in the egg could reduce tissue tropism in later stages and therefore decrease the number of host cells in which *Wolbachia* can replicate. Finally, phage WO may be more susceptible to induction in the egg stage. Increased transcription of the phage in mosquito eggs relative to other tissues supports this notion that the phage may be most active or susceptible to activity during the egg stage [36].

Although the observed changes in bacterial and phage densities are qualitatively consistent with the hypothesis that lytic phage prey on *Wolbachia* and cause bacterial mortality, these dynamics may also be incidental to the heat-killing mechanism. For example, the decline in endosymbiont concentration could be entirely due to a hyperactive immune response in heat-reared wasps or bacterial death caused by the heat. The consequent reduction of endosymbiont numbers in such scenarios, without a concurrent reduction in phage WO particles, might fully account for the relative increases in phage densities and the declines in *Wolbachia* densities. In this interpretation, the heat directly drives the changes in the bacterial populations, rather than the heat driving the phage population, which in turn drives the changes in the bacterial population. Experiments comparing phage-containing and phage-free *Wolbachia* under heat treatment are necessary to completely rule out this alternative; however, there are no such polymorphisms for phage WO in *Wolbachia*-infected hosts. As a result, we are currently engineering a phage-free *Wolbachia* into a phage-containing one in the laboratory.

If temperatures selectively induce phage WO, one question that must be considered is how is it done. The bacterial SOS system is comprised of a suite of genes aimed at preserving cell survival in the presence of extensive DNA damage [37]. The SOS response is induced by the activation of the RecA protein, whose gene is present in *Wolbachia* [6], after binding to single-stranded DNA fragments [38]. Activated RecA then promotes the autocatalytic cleavage of the LexA repressor in *Escherichia coli* in addition to several lytic cycle repressors of temperate bacteriophages [37], [39]. The induction of lytic development in *Wolbachia* may be allied with the SOS response [22]. Key SOS genes are present in the *Wolbachia* genome, including *recA*, *uvrA*, *uvrB*, *uvrD*, and a cI-like phage repressor; the *lexA* repressor is missing. Furthermore, electron micrographs in *Nasonia* testes indicate a massive degradation of the *Wolbachia* chromosome during the development of the bacteriophage lytic cycle [8]. The degraded DNA may serve to induce the SOS response, leading to lytic development of the bacteriophage. Increased phage production can then have a positive feedback since phage particles released from its resident cell could potentially attach to and lyse other *Wolbachia* coinfections or transfer into recipient cells that lack the phage, thus making these recipient cells susceptible to future development of lytic bacteriophage. There is extensive evidence for phage transfer between *Wolbachia* coinfections in the same host in both laboratory strains [2], [4], [16] and field populations [40].

Studies on *Wolbachia* dosage also help to elucidate how the bacteria may interact with the host to induce reproductive parasitism. During the course of measuring *Wolbachia* densities in these experiments, we observed the typical, positive relationship between bacterial dosage and CI expressivity within experimental treatments (Figure 6). However, between different temperature treatments, there was a decoupling of *Wolbachia* densities and CI expressivity. For instance, when the 18 C cold treatments showed a slight but significant decrease in *Wolbachia* densities relative to the 25 C control, there was a corresponding increase in CI levels (Figure 5A). Similarly in the 30 C heat-reared samples from the egg stage that showed a strong, significant decline in *Wolbachia* densities, there was no change in CI expressivity compared to the 25 C control group (Figure 5B). In addition, the heat-treated adult stage with significantly lower CI did not have significantly lower *Wolbachia* densities. This decoupling of the bacterial dosage model of CI between experimental treatments is perhaps unexpected. However, in a previous study in *Leptopolina heterotoma* wasps, males reared at different temperatures expressed the same levels of CI, despite their different densities [32]. One explanation for this observation is that the infection densities at various temperatures in *L. heterotoma* wasps exceeded a threshold density to induce full CI. This hypothesis does not completely apply to our results as males with lower CI did not necessarily have lower densities compared to the 25 C control.

How can these results be explained? Different temperature regimes could alter multiple variables in the induction of CI including host development time, host cell number, and bacterial tissue tropism. For instance, density measurements were recorded in whole adults in this experiment, so the variation in densities between treatments may be confounded by differences in somatic cell densities of the infection. We also found that the dosage threshold to induce complete CI actually decreases with the duration of the developmental period exposed to temperature. For example, cold-reared samples from the egg stage expressed more complete CI than cold-reared samples from the larval stages, and so on. The statisical results also indicate that cold-reared samples from egg and larvae, and heat shocked samples from adulthood, are the the only three stages in which CI was altered in comparison to the 25 C controls. It was increased in the cold stages and decreased in the heat-treated adults. As there is no apparent microbial variable, such as *Wolbachia* or phage WO densities, that change in parallel with these CI differences, it appears that microbial densities do not explain the variation across treatments. One significant variable is that the length in development time from oviposition to adult emergence is 30 days, 14 days and 10 days when reared at 18 C, 25 C and 30 C, respectively. A longer development time in the cold increases CI and a short heat exposure in adulthood reduces CI. A notable aspect of spermatogenesis in *Nasonia* is that it is synchronized within a testis. While the timing of the sperm modificaiton remains elusive, heat shock of third instar larvae in *Drosophila* leads to reduced CI, suggesting that the CI modification can be altered even up until spermatid elogation in mature sperm cysts in this system [19]. Since *Nasonia* spermatognonia and spermatocytes are visible at the early pupal stages [41], with mature sperm forming in late pupae, there is a critical juncture in development between the larval and pupal stages. Thus, if *Wolbachia*'s modication is induced before the pupal stage in stem cells, or early spermatogonia and spermatocytes, then perhaps the slower *Nasonia* development in the 18 C cold treatment allows *Wolbachia* more time to modify these cells for full induction of CI. In contrast, in the heat-reared adults, the CI modification may have been disrupted. Since *Wolbachia* are not in all spermatids and there is a scattered distriubtion of the *Wolbachia* throughout the testes, it may be difficult for *Wolbachia* to re-modify the CI-inactivated sperm. Future work which links the induction of CI to the developmental stage of sperm would help elucidate these issues.

We conclude with a summary of the key findings described in this study: First, 18 C and 30 C are temperatures at the edges of *Nasonia*'s habitable range and both decrease *Wolbachia* densities. Second, the declines in *Wolbachia* correlate with increases in phage densities, and heat expectedly has the marked effect on increasing phage densities. Third, there was an effect of insect family on phage WO and endoysmbiont densities. Offspring derived from the same mother had more similar variation in microbial densities than they did to offspring derived from other mothers, suggesting there may be maternal or other micro-environmental effect on microbial densities of *Wolbachia* and phage WO. Finally, there was an interesting temperature-mediated adjustment to the density threshold at which *Wolbachia* cause complete CI, which led to a decoupling of the relatioship between bacterial densities and CI penetrance between treatment groups with different temperatures.

## Supporting Information

Table S1 Absolute Copy Numbers of *NvS6K*, *groEL* and *ORF7* genes per *N. vitripennis* male.
(XLS)
